# Player Monitoring in Indoor Team Sports: Concurrent Validity of Inertial Measurement Units to Quantify Average and Peak Acceleration Values

**DOI:** 10.3389/fphys.2018.00141

**Published:** 2018-02-27

**Authors:** Mareike Roell, Kai Roecker, Dominic Gehring, Hubert Mahler, Albert Gollhofer

**Affiliations:** ^1^Department for Sports and Sport Science, Albert-Ludwigs-University Freiburg, Freiburg im Breisgau, Germany; ^2^Applied Public Health, Furtwangen University, Furtwangen im Schwarzwald, Germany

**Keywords:** locomotion analysis, orientation estimation, inertial measurement unit, complementary filter, physical demands, indoor team sports

## Abstract

The increasing interest in assessing physical demands in team sports has led to the development of multiple sports related monitoring systems. Due to technical limitations, these systems primarily could be applied to outdoor sports, whereas an equivalent indoor locomotion analysis is not established yet. Technological development of inertial measurement units (IMU) broadens the possibilities for player monitoring and enables the quantification of locomotor movements in indoor environments. The aim of the current study was to validate an IMU measuring by determining average and peak human acceleration under indoor conditions in team sport specific movements. Data of a single wearable tracking device including an IMU (Optimeye S5, Catapult Sports, Melbourne, Australia) were compared to the results of a 3D motion analysis (MA) system (Vicon Motion Systems, Oxford, UK) during selected standardized movement simulations in an indoor laboratory (*n* = 56). A low-pass filtering method for gravity correction (LF) and two sensor fusion algorithms for orientation estimation [Complementary Filter (CF), Kalman-Filter (KF)] were implemented and compared with MA system data. Significant differences (*p* < 0.05) were found between LF and MA data but not between sensor fusion algorithms and MA. Higher precision and lower relative errors were found for CF (RMSE = 0.05; CV = 2.6%) and KF (RMSE = 0.15; CV = 3.8%) both compared to the LF method (RMSE = 1.14; CV = 47.6%) regarding the magnitude of the resulting vector and strongly emphasize the implementation of orientation estimation to accurately describe human acceleration. Comparing both sensor fusion algorithms, CF revealed slightly lower errors than KF and additionally provided valuable information about positive and negative acceleration values in all three movement planes with moderate to good validity (CV = 3.9 – 17.8%). Compared to x- and y-axis superior results were found for the z-axis. These findings demonstrate that IMU-based wearable tracking devices can successfully be applied for athlete monitoring in indoor team sports and provide potential to accurately quantify accelerations and decelerations in all three orthogonal axes with acceptable validity. An increase in accuracy taking magnetometers in account should be specifically pursued by future research.

## Introduction

Knowledge about physical demands in team sports has become increasingly important to optimize training programs, to enhance physical performance and to prevent injuries (Fox et al., 2017; Vanrenterghem et al., 2017). Several monitoring systems have been developed to simultaneously quantify multiple players' position, velocity and acceleration during sport specific locomotion (Chambers et al., 2015; Li et al., 2016). In order to rely on a monitoring system's output for player monitoring, data should be both valid and reliable. It is important to note that high consistency in measurements of a system indicates its ability to determine evident and meaningful changes in an athlete's performance. The amount of error caused by high variability within or between monitoring tools as well as the agreement between measured and true values should therefore always be taken into account when performance is assessed and evaluated. Especially, GPS-based systems have recently been evaluated as applicable monitoring tools and are commonly applied in outdoor sports (Cummins et al., 2013; Johnston et al., 2014). Reduced signal quality, however, disables their usage in indoor environments. Alternatively, indoor monitoring systems have been developed and can be subdivided into vision-based or microtechnological systems. Although vision-based motion analysis is widely applied for player monitoring, findings about validity and reliability are inconsistent due to the multitude of existing systems and their dependency upon manual intervention, quality of video footage or camera positioning (Duthie et al., 2005; Barris and Button, 2008). Permanently installed microtechnological local positioning systems are able to overcome these problems showing high values of reliability (CV <2%) and validity with reported typical errors of 1.2-9.3% for distance, speed and acceleration (Leser et al., 2014; Rhodes et al., 2014; Serpiello et al., 2017). However, mentionable errors were found for mean and peak deceleration (TE_mean_ = 84%, TE_peak_ = 20%) as well as a decrease of accuracy for actions at the side of the court (Serpiello et al., 2017). Furthermore, high costs and local restrictions due to their fixed installation limit the application of local positioning systems (Hedley et al., 2010; Stevens et al., 2014). Lately, the technological development of inexpensive and portable Micro Electro Mechanical Systems (MEMS) enabled possibilities of quantifying physical loads with a robust method even during games or training sessions in different sports facilities. Most sport specific tracking devices nowadays include a 9 degree of freedom triaxial inertial measurement unit (IMU) containing an accelerometer, gyroscope and magnetometer within a GPS tracking device. Applied in indoor environments these devices enable sampling of acceleration-based data in high resolution during sporting activities without the support from GPS-signals. Extracting sports relevant data from the sensor's signals indoors without the aid of external references is complicated due to multiple sources of noise, mainly by earth's gravity acceleration. Several approaches were proposed therefore to estimate the tracking device's orientation with respect to the earth's coordinate system, e.g., sensor fusion algorithms without GPS (Madgwick et al., 2011; Sabatini, 2011a; Valenti et al., 2015). Such algorithms commonly combine accelerometer and gyroscope signals to compute the device's attitude (pitch and roll angles) relative to the direction of gravity. Including magnetometer readings into the algorithm enables the computation of the sensor's heading, meaning its deviation from magnetic north. Highly dynamic changes of the device's orientation as they frequently occur during sporting activities challenge an algorithm's accuracy. As accepted standard, stochastic Kalman-Filter-based techniques (KF) are commonly applied as effective tool in human motion analysis (Sabatini et al., 2006; Sabatini, 2011a), giving a probabilistic determination of modeled state estimations with the goal of minimizing errors from the true value. Due to multiple tuning parameters their main advantage lies in a high accuracy and a broad field of applications exceeding the purpose of orientation estimation. Complementary Filters (CF) serve as frequency-based equal alternative because of their algorithmic simplicity, effective performance and less difficult implementation process. Due to the dependency upon the single sensors' frequency characteristics the potential applications of CF are restricted, but provide equal and accurate results for orientation estimation (Madgwick et al., 2011; Tian et al., 2013; Valenti et al., 2015). Quantitatively, the time required for necessary linear regression iterations in KFs results in a slower convergence compared to CFs (Ricci et al., 2016). Considering the high frequency of movement changes observed in court-based team sports (Abdelkrim et al., 2007; Luteberget and Spencer, 2017) leading to consequently frequent changes of the device's orientation, the immediate convergence that was found for CFs might serve as appropriate foundation to provide accurate orientation estimation in indoor team sports. Although, CFs have not been evaluated thoroughly especially if compared to KF-based techniques for sport specific purposes, their effectiveness has already been proven for the analysis of human movements (Bachmann et al., 2001; Tian and Tan, 2012; Tian et al., 2013). Validity of a commercially available IMU-based monitoring system that relies on KF-techniques have been proven regarding the magnitude of the resulting acceleration vector or the instantaneous rate of change of acceleration (Wundersitz et al., 2013, 2015a,b). Based on those parameters activity profiles and quantification of loads during games and training have been proposed for indoor team sports (Montgomery et al., 2010; Schelling and Torres, 2016; Luteberget and Spencer, 2017). More elaborated discriminant analysis, however, is lacking because continuous information about gravity-corrected accelerations in the global anterior-posterior, lateral and vertical directions are typically not provided by manufacturers. Exact coordinates of the resulting vector with respect to the earth's coordinate system, however, would be desirable for profound game analyses, as they are already standard in outdoor team sports, leading to a deeper understanding of physical and underlying physiological demands. Recently a new approach has been proposed describing the relation between power output and time duration of movement as a general function for GPS-based analyses of soccer games (Roecker et al., 2017). The function is independent of arbitrary or experience-based intensity thresholds which offers apparently the transfer to acceleration-dominant indoor-sport analyses with the use of IMUs and appropriate sensor fusioning. Added value could be provided through additional information regarding the amount and intensity of acceleration components in the global x-, y- and z-direction as well as distinction between positive and negative acceleration. On this basis, interpretation of individual locomotion might be beneficial for individualization of training programs, supervision of rehabilitation processes or control of each player's injury risk.

The aim of the current study was to compare the concurrent validity of a recently published CF algorithm with a KF, provided by the sensor's manufacturer and a low-pass filtering method applied to IMU signals to obtain average and peak acceleration values in all movement planes. Data recorded from an IMU-based tracking device during simulated team sport specific movements is set against a 3D motion capture system.

## Materials and methods

### Preliminary investigation

In order to use IMUs for the purpose of orientation estimation, data output should be not only valid but also reliable. A number of studies already evaluated the tracking device that has been used in this study (Optimeye S5, Catapult Sports, Melbourne, Australia) regarding the accelerometer's intra- and inter-device variability under laboratory but also field conditions in handball, ice hockey and Australian football (Boyd et al., 2011; Luteberget et al., 2017; van Iterson et al., 2017). Coefficient of variation (CV) values well below the according smallest worthwhile difference were found during dynamic, mechanical motion (CV_inter_ < 1.04%; CV_intra_ < 1.05%) and sporting activities performed by subjects in the laboratory (CV_inter_ < 6.7%) or field (CV_inter_ < 2.1%; CV_intra_ < 26.6%). While these results indicate a good within- as well as between-device reliability of accelerometers, evidence regarding the gyroscope's reliability is missing. As the gyroscope's data output is critical for accurate orientation estimation, we evaluated within- and between-device reliability using a platform rotating at constant angular velocity of 199°/s and 270°/s respectively. A device mounted on the platform was rotated around either its x-, y-, or z-axis. 10 consecutive trials of 30 s rotation were recorded in each axis for overall 8 devices at both 199°/s and 270°/s. Between trials the turntable was standing still for 30 s. In both conditions a CV <1% was found for mean and peak angular velocity within as well as between devices, indicating an excellent reliability of the gyroscope. Overall, both accelerometer and gyroscope contained within the tracking device show low intra- and inter-device variability, indicating sufficient reliability of the underlying technology. As the output of the single inertial sensors can be equated with the sensor fusion algorithms' input, the applied tracking device can be stated to be reliable enough for further validation research.

### Procedure

Data of a single wearable tracking device including an IMU with a sampling frequency of 100 Hz (Optimeye S5, Catapult Sports, Melbourne, Australia) was compared to the results of a 3D motion analysis (MA) system operating at 200 Hz (Vicon Motion Systems, Oxford, UK) during several standardized movement simulations in an indoor laboratory.

To eliminate unintentional artifacts, the device was clamped into a stiff wooden frame that was adapted to the dimensions of the device. The investigator manually moved the frame according to predefined movement simulations inside the capturing volume of the MA system. The simulations were chosen to imitate orientations and changes of orientation as they would equally occur during team sport specific movements. Constant mono- or multi-planar motion of the investigator was combined with different orientations of the device including rotations around x-, y-, and z-axis between or during each trial (Table 1). Prior and after each trial, the frame was stroked against the ground to evoke a trigger signal for synchronization of the IMU and the MA system. Each of the 28 movement simulations was performed and recorded two times within one recording session. The device has not been turned on and off between trials to simulate long-term usage as it would also appear during training sessions or games. Calibration has been performed by the manufacturer and was therefore not repeated manually prior to recording.

**Table 1:** Summary of recorded team-sport specific movements during data collection.

	**Sensor movement**	**Sensor orientation**	**Imitated movement**		**Sensor movement**	**Sensor orientation**	**Imitated movement**
Standardized movements	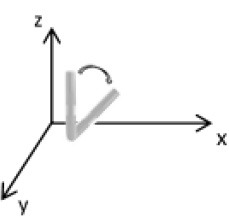	(1)	Leaning forward	Walking, running	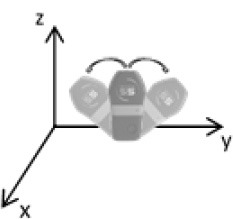	(1)	Walk forward
						(3)	–
						(2)	Inclined torso position during walking
						(1)	Sprint forward
						(4)	Lateral movement in forward direction (i.e., sidesteps/shuffle)
						(2)	Inclined torso position during sprinting
	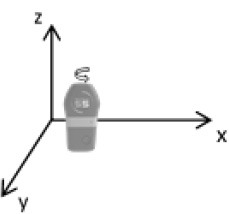	(4)	Leaning sideways			(3)	–
						(1), (4) alt.	Change of orientation during walking
						(4) w/45° tilt	Inclined torso position during side movement
						(1) w/ 180° rotation	Forward walking into backwards running
	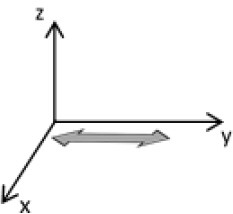	(1)	Upper body rotation	Jumping	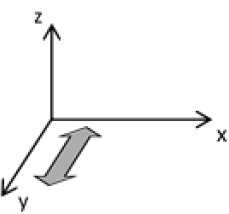		
						(1)	Vertical jump
						(2)	Vertical jump with inclined torso position
						(1)	Falling to the ground
Translational movements	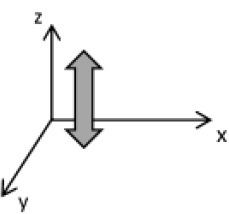	(1)	Anterior-posterior change of direction movement	Complex movements	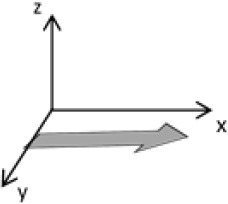	(1)	90° change of direction movement
		(4)	Lateral movement in anterior-posterior direction				
		(2)	Sprint forward and backpedal				
		pos. (4), neg. (1)	Lateral movement in positive direction and backwards running in negative direction				
	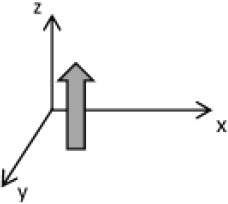	(1)	Lateral change of direction movement (straight motion)		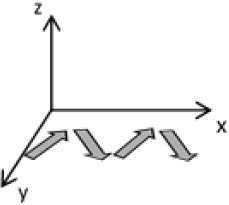	(1)	180° change of direction movement
		(1)	Lateral movement i.e. during defense (semicircle motion)				
	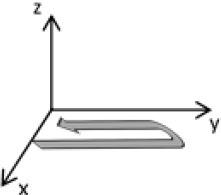				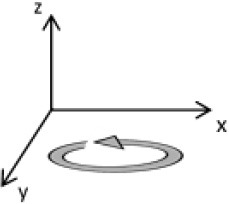		
		(1)	Jumping and landing (straight motion)			(1)	Constant walking over 1.5 min
		(1)	Jump to e.g., basket (semicircle motion)			(1), (2), (3), (4)	Combination of game specific movements (jumps, backwards, lateral, sprint, walk, etc.) over 1.5 min
**Sensor orientation with respect to reference coordinate system**							
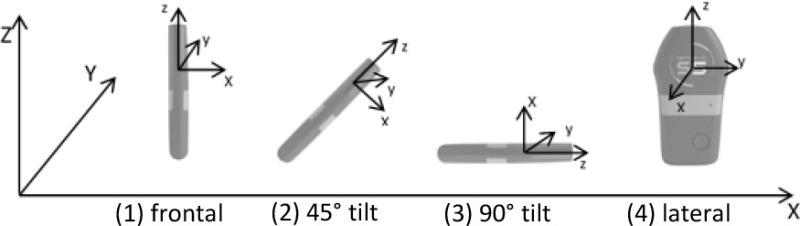							

Common orientations occurring during team sports were manually simulated.Intensities varied between movements from slow (walking) to fast (sprint, jump) to cover intensities that would equally occur during training or game. Observed absolute acceleration values covered a wide range of intensities varying from smallest values found ~1 m^*^s^−2^ to highest values ~21 m^*^s^−2^. Each trial was performed two times (n = 56).

Three retro-reflective markers (Ø 14 mm) were attached to the edges of the rectangular wooden frame to capture the device's local coordinate system (LCS) optically and to calculate a single virtual marker at the estimated position of the IMU's sensor position within the dimensions of the tracking device. Calculation of the virtual marker was done with a custom written script (Bodybuilder, Vicon Motion Systems, Oxford, UK). These virtual marker's trajectories were used for further analysis.

For the purpose of our study, ethical approval and written informed consent were not mandatory since it neither contained human subject research nor recruitment of human subjects, physical or psychological interventions or clinical research practices. Movements of the IMU in the laboratory were performed by the investigator, being well aware of the executed simulation movements. At no point of data collection any risks concerning the investigators physical or psychological health were apparent.

### Data processing

Raw data for both the MA system and the IMU were exported to Microsoft Excel (Microsoft Excel 2013, Version 15.0, Redmond, USA) through the according manufacturer-supplied software (Nexus 2, Vicon Motion Systems, Oxford, UK; Sprint 5.1.7, Catapult Sports, Melbourne, Australia).

After frequency-reduction of the MA system data to 100 Hz (Biomechanics Toolbar Version 1.02, Liverpool John Moores University, UK) a fourth order, zero-lag, low-pass digital Butterworth-Filter was applied to reduce noise from the x, y and z positional data. According to a residual analysis (Winter, 2009) an optimal cut-off frequency of 5 Hz was chosen. Due to a standard deviation (SD) <1.0 Hz between all trials the same cut-off frequency was applied to all trials. To exclude phase-shift dual pass filtering and a correction of the cut-off frequency to 6.23 Hz was applied (Winter, 2009). Acceleration values (m^*^s^−2^) in all three orthogonal planes were calculated through double numerical differentiation of the smoothed data. Data of the accelerometer (g) were converted to m^*^s^−2^ and data of the x and y-axes inverted once due to the tracking device's orientation within the wooden frame.

#### Low-pass filter (LF)

As first option to separate gravity from the sensor's readings a traditional method for gravity correction was applied. Relying on the assumption that gravitational signals only contain low-frequency components body-induced and gravity-induced accelerations can be separated (Bartlett, 2013; Mönks, 2017). Through low-pass filtering (LF) the acceleration data in each axis with a cut-off frequency of 0.3 Hz (Butterworth 4th order), the constant earth's gravity vector was extracted and afterwards subtracted from original acceleration values. The resulting signal was smoothed (Butterworth 4th order) using a cut-off frequency of 5 Hz (6.23 Hz corrected) for all trials (*SD* < 1.0 Hz) after visual inspection of residual analysis outputs (Winter, 2009) and smallest mean bias to MA reference data.

#### Complementary filter (CF)

**Figure 1:**
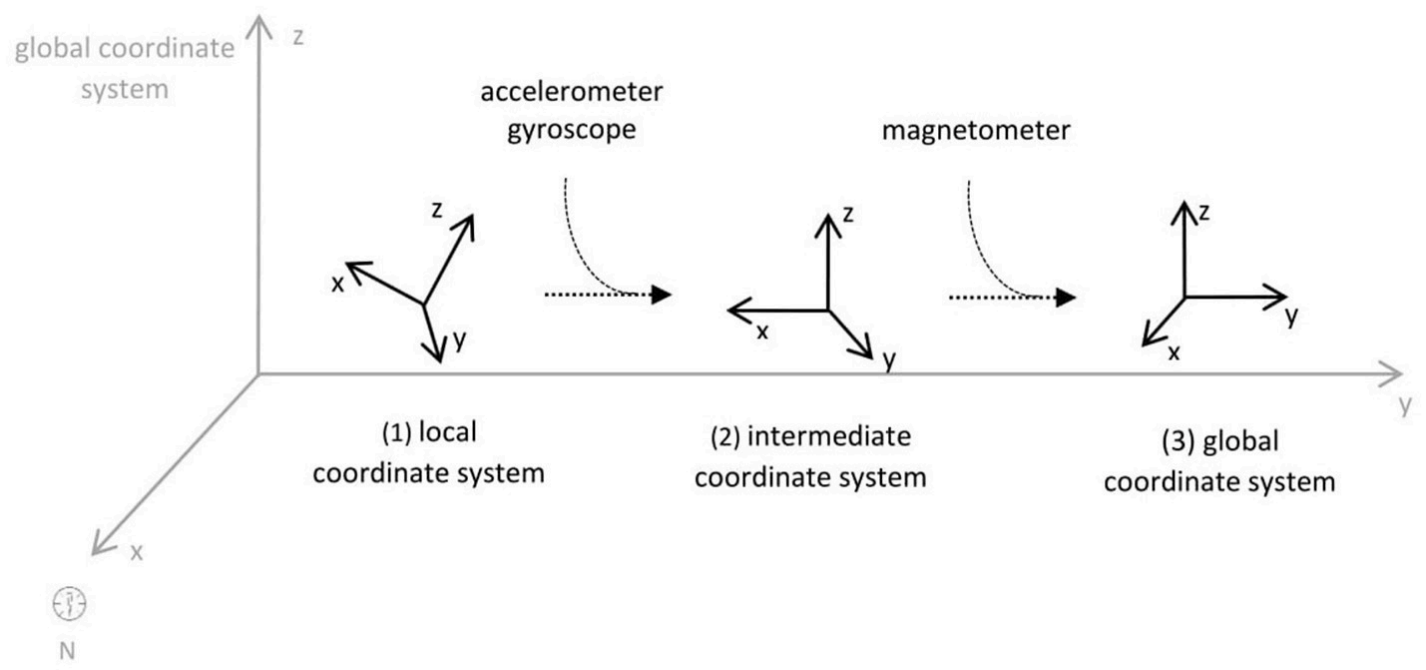
Rotations performed by the CF algorithm with respect to the reference system. The coordinate system in gray represents the GCS with the x-axis pointing toward magnetic north. (1) Represents the orientation of the IMU within the GCS. (2) Result of sensor fusing accelerometer and gyroscope data. The horizontal axes are parallel to the earth's surface but rotation about the z-axis is missing. Including magnetometer data to the calculations results in (3) with the x-axis of the previous LCS being aligned with the GCS. For the aim of this study the LCS (1) has only been rotated into the intermediate coordinate system (2).

As second option a sensor fusion algorithm (CF) that was originally developed to navigate unmanned aerial vehicles (Valenti et al., 2015) was implemented and adapted to human motion. These algorithms determine the orientation of the tracking device's LCS with respect to the global coordinate system (GCS) using a quaternion based approach. The applied CF has been developed to estimate the device's absolute orientation in two consecutive steps. In the first step, accelerometer and gyroscope data are used to correct the LCS for tilt. Through low-pass filtering the accelerometer signal and high-pass filtering the integrated gyroscope readings with the same cut-off frequency the complementary filter creates “complement” signals that are fused together to estimate the sensor's orientation. The resulting intermediate coordinate system with x- and y- axes being planar to the GCS represents the computed attitude estimation as relative orientation. The algorithm enables the estimation of an absolute orientation in a second step by correcting the intermediate coordinate system's yaw angle. This second step is only performed if magnetometer data are included in calculations and results in a GCS with the positive x-axis always pointing toward magnetic north (Figure 1).

To compare inertial data with the MA system, only the first step of the proposed CF was implemented to calculate the device's relative orientation as the MA system's x-axis has not been aligned to magnetic north during calibration. The cut-off frequency for the accelerometer data is constantly characterized using an adaptive gain algorithm within the CF. An initial filtering gain of 0.0072 was chosen, which is based on another CF that has been applied in human motion analysis (Tian et al., 2013). Accelerometer readings were converted to m^*^s^−2^ and multiplied with the quaternion of attitude estimation to rotate x-, y-, and z-vectors into the intermediate coordinate system. Constant gravitational acceleration was removed by subtracting 9.81 m^*^s^−2^ of the intermediate z-vector. All calculations were performed using routines written in C++ (compiled and edited with Microsoft Visual C++ 2017, Redmond, USA). Resulting acceleration vectors were then low-pass filtered (4th order Butterworth) with a cut-off frequency of 5 Hz (6.23 Hz corrected; *SD* < 1.0 Hz). The cut-off frequency has been determined due to the lowest mean bias between CF data filtered at cut-off frequencies from 4 to 10 Hz and the MA system. Due to the tracking device's orientation within the wooden frame the resulting acceleration values in x- and y-axes had to be inverted once.

#### Kalman-filter (KF)

As current standard for sensor fusion, a Kalman-Filter (KF) has not been implemented explicitly since it is provided by the manufacturer's software (Sprint 5.1.7, Catapult Sports, Melbourne, Australia). The manufacturer's results were chosen since they have previously been validated and are known to be designed specifically for sport specific environments (Wundersitz et al., 2013, 2015b). The manufacturer's software provides one continuous Kalman-filtered parameter, which is the magnitude of the resultant vector representing the combined effects of x-, y- and z-vectors corrected for gravity. This variable has been exported to Microsoft Excel for further analysis and was low-pass filtered for reducing unwanted noise (4th order Butterworth). 8 Hz (9.97 Hz corrected) has been chosen as cut-off frequency for all trials after residual analysis (*SD* < 1.0 Hz) and lowest mean bias compared to the MA system criterion. The same parameter has been calculated for MA system data as well as for LF and CF data.

### Data analyses

After data processing CF, KF, LF, and MA system data of each trial were synchronized by overlaying peaks of the triggering signals. Trigger signals were then excluded for further analysis so that only movement sequences were included. For each trial the average magnitude of the overall acceleration (total_x/y/z_), positive acceleration (acceleration_x/y/z_), and negative acceleration (deceleration_x/y/z_) as well as the peak magnitude of positive and negative acceleration values were calculated for CF, LF, and MA system data in x-, y-, and z-axes. Average magnitude as well as peak magnitude of the resultant vector (resultant_x/y/z_) were calculated for CF, KF, LF, and MA system data. To assess the agreement between IMU-based variables and MA system variables mean bias, root mean square error (RMSE; Barnston, 1992), 95% limits of agreement (Atkinson and Nevill, 1998), Spearman's correlation coefficient and the percentage difference in the mean between criterion (MA) and measurement (CF, KF, LF) expressed as coefficient of variation (CV; Hopkins, 2000) were calculated for mean and peak acceleration values in x, y and z-axes (CF, LF, MA) as well as average and peak magnitude of the resulting vector (CF, KF, MA). According to previous research evaluating the relative error of IMU-based acceleration variables a CV ≤5% was considered as small, CV ≥5% and <20% as moderate and CV ≥20% as large (Wundersitz et al., 2015b; Alexander et al., 2016). To approve the acceptable use of MEMS-based sensors in the field a CV <20% was intended (Tran et al., 2010; Wundersitz et al., 2015a).

### Statistical analyses

All statistical analyses were performed using JMP Version 13.1.0 (SAS Instituts Inc., Cary, NC, USA). Data are presented as mean ± *SD* with statistical significance set at *p* ≤ 0.05 except otherwise stated. Shapiro-Wilk-Tests revealed heteroscedastic data sets for mean and peak accelerations and magnitude variables. Therefore, a nonparametric one-way ANOVA on ranks (Kruskal-Wallis test) was applied to determine differences in mean and peak variables between CF, KF, LF, and MA system data. Mann-Whitney-*U* tests were additionally performed *post-hoc* to determine if differences in the means of measurement systems were evident for each variable. The α-level was adjusted to α = 0.017 after Bonferroni-correction to compare mean and peak magnitude acceleration values in all three axes between CF, LF and MA data. To identify differences in mean and peak magnitude values of the resulting vector between CF, KF, LF and MA system α-level was set at α = 0.013 after Bonferroni-correction. Effect sizes (r) for all performed statistical tests were calculated and interpreted according to Cohen (1992). Bland-Altman plots for all CF mean and peak variables against the MA system were used to visually evaluate the CF data in all axes (Bland and Altman, 1999).

## Results

Regarding average acceleration, significant differences were found between CF, LF and MA system data for total_y_, acceleration_x_, acceleration_y_ and deceleration_y_ (*p* < 0.05; r = 0.12 – 0.87; Table 2). Peak values showed significant differences in acceleration_x_, acceleration_y_ and deceleration_y_ (*p* < 0.05, r = 0.10 – 0.26). *Post-hoc* Mann-Whitney-*U* tests revealed, that without orientation estimation the gravity component could not accurately be eliminated in all three axes leading to significant differences for total_y_, mean/peak acceleration_y_, mean/peak deceleration_y_, peak deceleration_x_ and peak deceleration_z_ between LF data and MA system (*p* < 0.017, *r* = 0.39 – 0.50), whereas no significant differences were found between CF data and MA system. The LF and CF method significantly differed regarding total_y_, mean/peak acceleration_x_, mean/peak acceleration_y_ as well as mean/peak deceleration_y_ (*p* < 0.017, *r* = 0.34 – 0.55).

**Table 2:** Analysis of agreement between CF data respective LF data and MA system data.

**Variable**	**CF data**					**LF data**				
	Mean bias ± *SD* (m^*^s^−2^)	95% LoA (m^*^s^−2^)	**r_s_**	RMSE (m^*^s^−2^)	**CV (%)**	Mean bias ± *SD* (m^*^s^−2^)	95% LoA (m^*^s^−2^)	**r_s_**	RMSE (m^*^s^−2^)	**CV (%)**
**MEAN**										
Total_x_	−0.02 ± 0.05	−0.13 to 0.09	0.98	0.06	8.0	0.09 ± 0.96	−1.80 to 1.97	0.52	0.96	75.0
Total_y_^*^	−0.01 ± 0.04	−0.08 to 0.07	0.97	0.04	7.7	0.28 ± 0.74	−1.18 to 1.74	0.37	0.79	58.5
Total_z_	−0.02 ± 0.15	−0.32 to 0.27	0.98	0.15	9.3	−0.11 ± 0.71	−1.50 to 1.27	0.70	0.71	48.4
acc_x_^*^	0.08 ± 0.15	−0.21 to 0.38	0.96	0.17	15.9	−0.18 ± 1.03	−2.20 to 1.83	0.53	1.03	77.6
acc_y_^*^	−0.01 ± 0.08	−0.17 to 0.15	0.96	0.08	9.7	−0.27 ± 0.78	−1.79 to 1.25	0.72	0.82	60.3
acc_z_^*^	−0.02 ± 0.22	−0.46 to 0.41	0.97	0.22	11.9	0.25 ± 0.81	−1.33 to 1.84	0.72	0.84	49.6
dec_x_^*^	−0.02 ± 0.11	−0.24 to 0.19	0.97	0.11	11.4	−0.02 ± 0.97	−1.92 to 1.89	0.45	0.96	76.8
dec_y_^*^	0.04 ± 0.06	−0.07 to 0.15	0.97	0.07	9.3	−0.30 ± 0.71	−1.70 to 1.11	0.69	0.77	57.6
dec_z_^*^	−0.03 ± 0.14	−0.24 to 0.31	0.97	0.14	8.7	−0.03 ± 0.69	−1.39 to 1.33	0.66	0.69	47.6
**PEAK**										
acc_x_^*^	0.26 ± 0.54	−0.80 to 1.32	0.97	0.59	17.8	−1.07 ± 3.45	−7.84 to 5.71	0.66	3.59	64.8
acc_y_^*^	−0.08 ± 0.38	−0.83 to 0.68	0.95	0.39	15.0	−1.10 ± 2.04	−5.09 to 2.90	0.66	2.30	55.0
acc_z_^*^	0.12 ± 0.36	−0.60 to 0.83	0.98	0.38	6.7	0.34 ± 1.76	−3.10 to 3.78	0.87	1.77	27.4
dec_x_^*^	0.04 ± 0.45	−0.85 to 0.93	0.96	0.45	13.2	−0.50 ± 3.99	−8.32 to 7.31	0.50	3.98	80.7
dec_y_^*^	0.21 ± 0.36	−0.51 to 0.92	0.95	0.42	15.6	−1.12 ± 2.03	−5.09 to 2.86	0.72	2.30	54.0
dec_z_^*^	−0.09 ± 0.22	−0.51 to 0.33	0.99	0.23	3.9	0.37 ± 1.24	−2.06 to 2.81	0.76	1.29	30.4

*Mean and peak acceleration values are presented for overall acceleration (total), positive acceleration (acceleration), and negative acceleration (deceleration) in x-, y- and z-axis*.

* *Significant differences in the mean between LF and MA (p < 0.017) SD, standard deviation; 95% LoA, 95% limits of agreement; r_s_, Spearman's correlation coefficient; RMSE, root mean square error; CV, coefficient of variation*.

**Figure 2:**
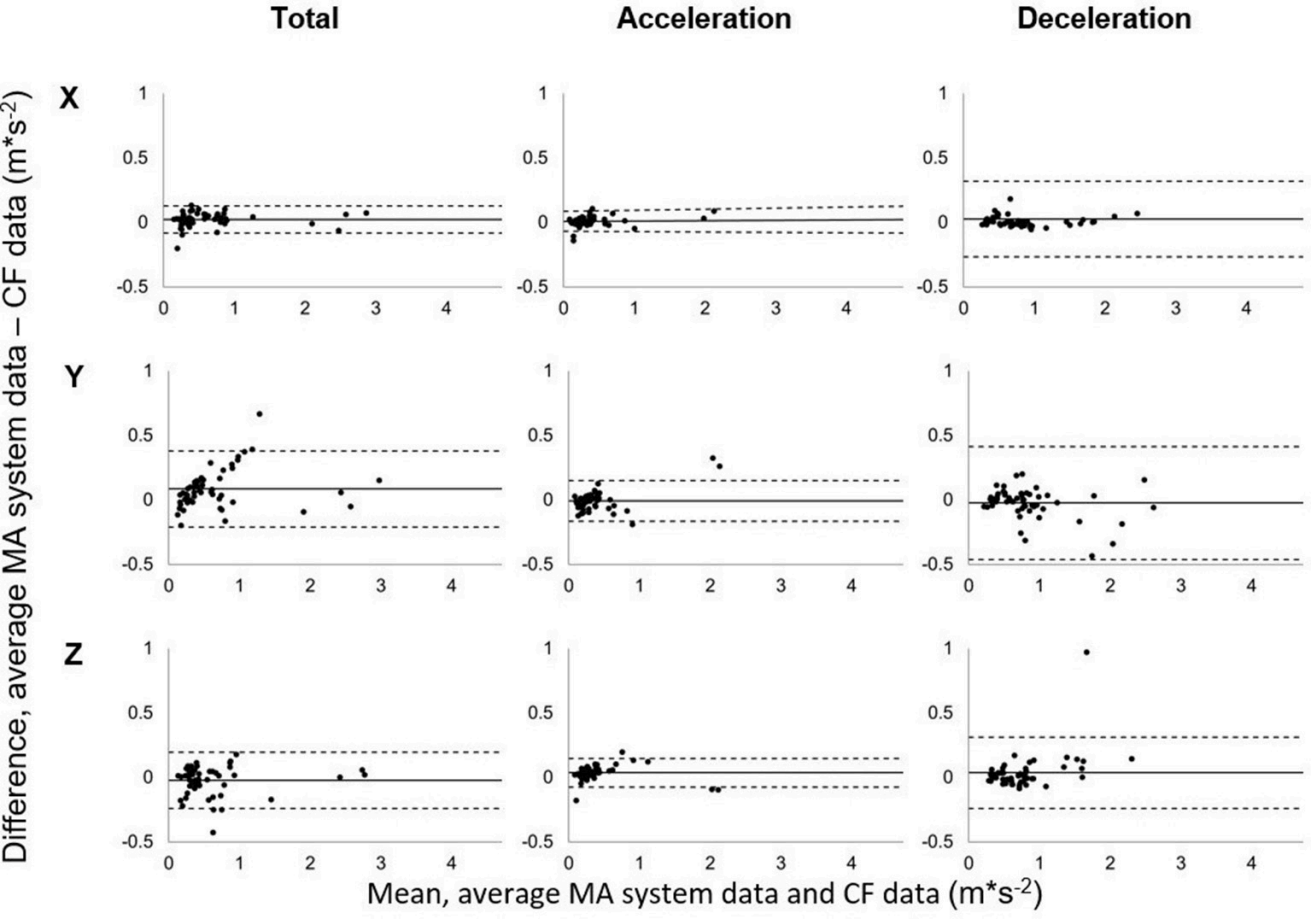
Bland-Altman plots showing the relationship between MA system data and CF data for average total, positive, and negative acceleration in x-, y-, and z-axis each. Dashed lines: 95% LoA, solid line: mean bias.

**Figure 3:**
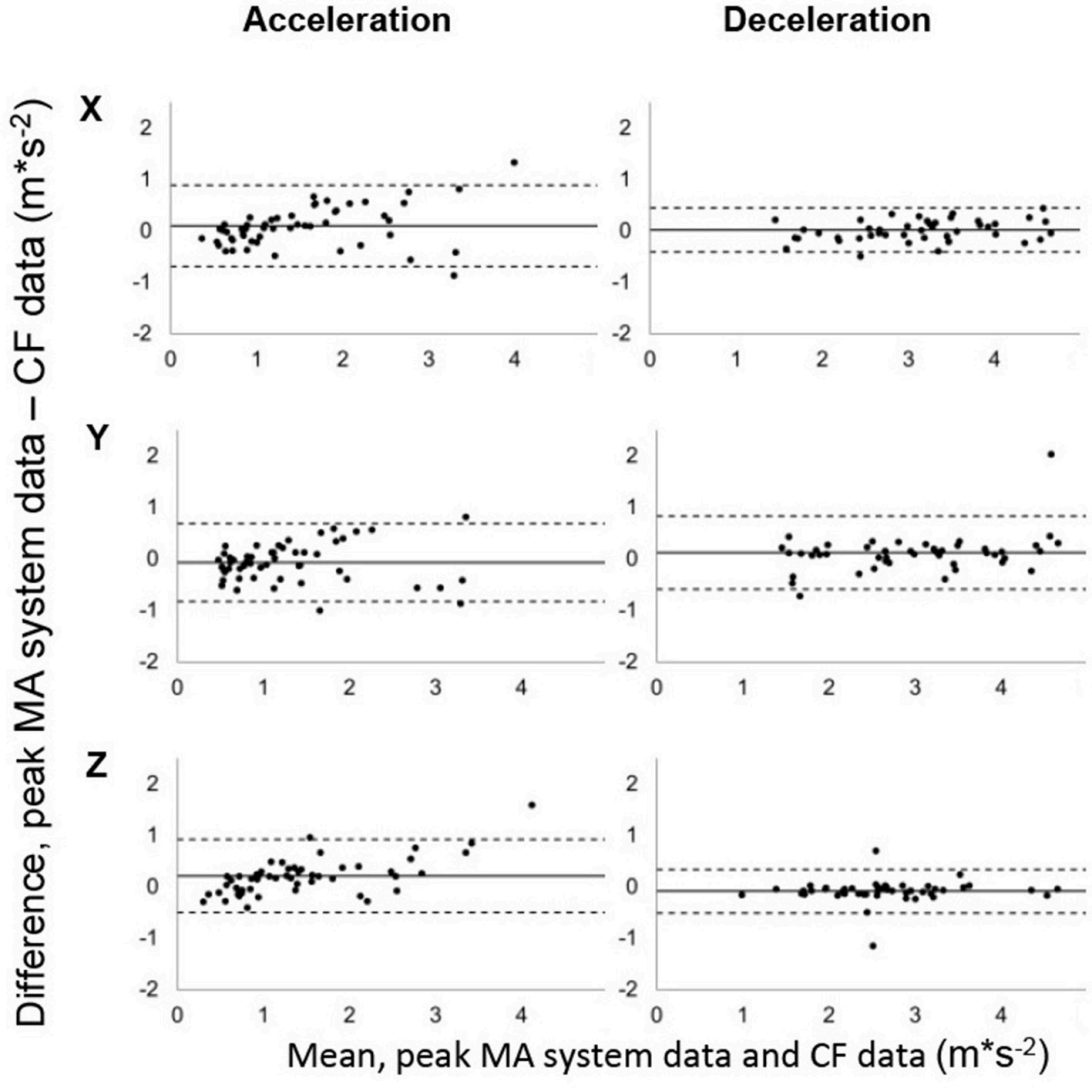
Bland-Altman plots showing the relationship between MA system data and CF data for peak total, positive, and negative acceleration in x-, y-, and z-axis each. Dashed lines: 95% LoA, solid line: mean bias.

Analysis of agreement support these findings with a high relative error regarding mean/peak acceleration values of the LF technique in x-, y- and z-axis (CV = 27.4 – 80.7%, RMSE = 0.37 – 0.72 m^*^s^−2^). LF method showed poor results also regarding accuracy, precision, correlation coefficient and limits of agreement (Table 2). Implementation of the proposed CF clearly improved measurement indices when compared to the LF data with a relative error of 8.0–15.9% for average magnitude values and 3.9–17.9% for peak magnitude values respectively. A low RMSE was found for mean acceleration values (RMSE = 0.04 – 0.22 m^*^s^−2^) whereas a higher error could be determined for peak values (RMSE = 0.23 – 0.59 m^*^s^−2^). Bland-Altman plots for mean acceleration values in all axes are shown in Figure 2 and indicate improved agreement of positive acceleration values compared to deceleration. No systemic bias could be observed for mean acceleration values as well as for peak acceleration values (Figures 2, 3). Limits of agreement exceed when regarding peak acceleration values in all axes (Figure 3) but still are within an acceptable range.

Comparison of the resulting vector's magnitude between CF, KF, LF, and MA system data revealed no significant differences for average as well as peak resulting magnitude values (*p* < 0.013). Although no significant differences could be found agreement analysis indicate poor accuracy, precision, limits of agreement and relative error of the LF method for mean and peak variables. Analysis of agreement for the results of both orientation filters (CF, KF) however indicate high accuracy in quantifying mean and peak resulting magnitude values. In contrast to the LF method low RMSE and CV values were found for CF and KF, indicating a high accuracy of both methods (Table 3). Thereby, slightly smaller errors of the CF data compared to the manufacturer's KF in all reported parameters could be noted.

**Table 3:** Analysis of agreement between KF data, LF data, CF data, and MA system data.

**Variable**	Mean Bias ± *SD* (m^*^s^−2^)	95% LoA (m^*^s^−2^)	**r_s_**	RMSE (m^*^s^−2^)	**CV (%)**
**MEAN**					
Resultant_KF_	0.11 ± 0.10	−0.08 to 0.30	0.99	0.15	3.8
Resultant_LF_	0.12 ±1.14	−2.12 to 2.35	0.61	1.14	47.6
Resultant_CF_	0.02 ± 0.05	−0.12 to 0.07	0.99	0.05	2.6
**PEAK**					
Resultant_KF_	0.52 ± 0.66	−0.78 to 1.81	0.98	0.83	7.1
Resultant_LF_	0.69 ± 2.08	−3.40 to 4.77	0.77	2.18	34.0
Resultant_CF_	0.01 ± 0.39	−0.78 to 0.76	0.99	0.39	4.9

*Mean and peak acceleration values are presented for the magnitude of the resulting acceleration vector*.

* *significant differences in the mean between data processing method and criterion (MA) (p < 0.013) SD, standard deviation; 95% LoA, 95% limits of agreement; r_s_, Spearman's correlation coefficient; RMSE, root mean square error; CV, coefficient of variation*.

## Discussion

### Main findings

Aim of this study was to evaluate the concurrent validity of two standard sensor fusion algorithms to accurately quantify and normalize team sport specific accelerations as well as decelerations under indoor conditions. Furthermore, it was intended to receive information alongside the resulting acceleration vector about positive and negative acceleration values in all three movement planes.

Our findings show that after implementation of a sensor fusion algorithm, the IMU-derived data do not substantially differ from the motion capture system data. Analysis of agreement indicate that the CF algorithm seems to be capable of quantifying average acceleration magnitude (CV = 8.0 – 15.9%, RMSE = 0.04 – 0.22 m^*^s^−2^) and peak acceleration magnitude (CV = 3.9 – 17.8%, RMSE = 0.23 – 0.59 m^*^s^−2^) in x-, y-, and z-axes within a good to moderate range. Validity could be shown for both sensor fusion algorithms regarding the magnitude of the resultant acceleration vector (Table 3). Although no differences were evident between CF and KF, slight advantages of CF were found according to analysis of agreement. Overall, MEMS-based tracking devices seem to provide promising information to continuously calculate human acceleration and deceleration through the application of adequate orientation filters and smoothing techniques even without the aid of external references.

### Comparison of LF, CF, and KF

Previous studies with relevance for team sports activities have reported that raw accelerometer data show insufficient accuracy as a measure of impacts during jumping movements or average acceleration during high-speed running (Tran et al., 2010; Alexander et al., 2016). The authors assumed these discrepancies to result from a lack of gravity-compensation. As IMU-based sensors are sensitive to all kinematic phenomena occurring within a time and space fixed inertial frame, earth's constant gravity and rotation is apparent in the sensor's reading. While earth's rotation with 15 degree/h compared to sensor noise is negligible for the current issue of interest (Sabatini, 2011b; Groves, 2013), a precise separation of human-induced accelerations and external bias, including earth's gravity is essential to accurately describe an athlete's locomotion. Our results indicate that the simple low-pass filtering to extract gravity-induced high-frequency components does not provide acceptable results (CV_mean_ > 20%, RMSE_mean_ = 0.69 – 1.03 m^*^s^−2^; CV_peak_ > 20%, RMSE_peak_ = 1.29 – 3.98 m^*^s^−2^). In contrast to sensor fusion techniques the exact direction of gravitational acceleration acting on the tracking device stays unknown, which seems to hinder accurate distinction between gravity and body acceleration. While the standard low-pass filtering method might be sufficient in primarily static environments, our results reveal serious errors when it comes to quantifying human acceleration during sport specific simulations including frequent orientation and movement changes. Contrastingly, both sensor fusion algorithms resulted in obvious improvements of accuracy and precision of the tracking data. Regardless of the filtering technique (stochastic vs. complementary) a high concurrent validity in measuring the resultant's vector magnitude was observed for mean and peak values. These observations strongly emphasize that future analysis must consider the orientation of the athlete in regard to the global coordinate system via sensor fusion. The KF-parameter provided by the manufacturer's software has previously been validated (Wundersitz et al., 2015a,b) during linear movements (CV = 6.5 – 9.5%) and a team sport specific circuit that included jumping, change of direction tasks and tackling (CV = 5.5%). Our findings support these results, indicating good to acceptable validity of the KF not only for quantifying peak (CV = 7.1%, RMSE = 0.83 m^*^s^−2^) but especially average values (CV = 3.8%, RMSE = 0.15 m^*^s^−2^) during a variety of team sports related movement simulations. Although both orientation filters show good results the applied CF slightly outperformed the KF regarding mean bias, limits of agreement, RMSE, correlation coefficient and relative error (Table 3). Bergamini et al. (2014) found errors in orientation estimation during locomotor trials depending on the task and type of orientation but independent of the type of sensor fusion. More detailed analyses under highly controlled conditions revealed slight differences occurring from the sensor fusion algorithm itself although the main dependency still resulted from the performed movement (Ricci et al., 2016). During dynamic trials with a robotic arm imitating human movements the implemented KF indeed showed an overall better performance but also a remarkably slower rate of convergence during static trials. While the CF immediately adapted to stops after a motion the KF technique required about 10 s to reach a stable signal. As in court-based sports movement changes occur about every 3 s (Abdelkrim et al., 2007; Luteberget and Spencer, 2017) and likely induce pauses of short duration, a faster rate of convergence might be beneficial in particular to follow these intermittent changes between highly dynamic motion and momentary stops. Since most of our trials were short of duration (<30 s) and included temporary pauses, e.g., during change of direction movements, the observed advantages of the CF could be explained by its faster adaption. However, we did not examine the algorithm's convergence rate directly. Furthermore, the properties of the manufacturer's algorithm are unknown while the choice of tuning parameters is critical for an algorithm's accuracy (Ricci et al., 2016). The exact reason for the discrepancies between the stochastic and complementary approach can therefore not be explained completely by our work.

### Analysis in movement planes

The proposed CF enables to overcome the restriction of analyzing primarily the magnitude of the resulting acceleration. Continuous discrimination in average acceleration and deceleration can be provided with moderate to good accuracy in all axes. For peak values, RMSE indicates a high accuracy in vertical direction, but an increase in the magnitude of the error for x- and y-values compared to average accelerations. Still, these values allow a good approximation of peak values within an error range of 0.39 – 0.59 m^*^s^−2^ and are lower than RMSE values found for resultant peak impacts in team sport movements (Wundersitz et al., 2015b). However, in addition with relatively high CV values practitioners should be aware of limited accuracy when analyzing single maximum values in both horizontal axes. Previous research examining the validity of MEMS-based sensors during sporting activities applied a maximum CV of 20% as limit for acceptable validity (Tran et al., 2010; Wundersitz et al., 2013, 2015a). Therefore, relative errors of 6.7% (acc_z_) and 3.9% (dec_z_) found in this study for peak acceleration values in the z-axis indicate good validity of the CF data. In the horizontal plane, RMSE values generally speak for the CF's validity and according relative errors could objectively be described as acceptable (CV < 20%). However, the measures in x- and y-axes cannot be stated to be accurate enough when quantifying especially high peak acceleration values. Relative errors up to 17.8% (acc_x_) could in fact equal errors >1 m^*^s^−2^ for high intense acceleration efforts (>6 m^*^s^−2^) and lead to large misinterpretations of a player's true performance. An internal non-orthogonality of the tracking device's axes could explain these findings, but is usually prevented with the use of calibration routines (Groves, 2013). For the purpose of this study, no calibration was performed prior to the recording as the manufacturer recommends to rely on the built-in calibration of the devices. More likely the described error results from a misalignment between the x- and y-axes of the computed intermediate frame with the reference axes of the MA coordinate system. Since no correction of the yaw-angle was performed deviations in the horizontal plane of the intermediate coordinate system with respect to the MA coordinate system could account for higher relative errors. This hypothesis is supported by the lowest CVs and RMSEs found for peak vertical parameters (RMSE_mean_ = 0.23 – 0.38 m^*^s^−2^, CV_peak_ = 3.9 – 6.7%) indicating a good correction of pitch and roll angles. It is assumed to reach similar values also for x- and y-axes if a perfect alignment of the calculated and the reference coordinate system is accomplished. However, our results show that the implementation of a complementary filtering technique results in a good level of validity when determining average and peak acceleration values in vertical direction as well as promising precision for the horizontal plane.

### Practical applications

Although accuracy should further be improved for horizontal and lateral direction our results suggest a successful application for developing discriminant acceleration-based activity profiles in indoor sports. Recent studies emphasize the importance of accelerations during team sports for the imposed external load on the athlete. Accelerations and decelerations are known for higher metabolic loads (Osgnach et al., 2010) and greater processes of muscular damage due to their eccentric loading (Nosaka and Newton, 2002; Lakomy and Haydon, 2004). Both could account for the decrease in acceleration efforts over time, observed in football matches which is assumed to indicate an increase in fatigue (Akenhead et al., 2013; Mara et al., 2017). A greater amount of physical loads including acceleration and deceleration based movements can be assumed for indoor court-based sports as an increase in physical demands and acceleration patterns has been observed with the reduction of pitch sizes during soccer games (Hodgson et al., 2014). Although the importance of this topic is widely accepted, only a limited number of studies examined the proportions of accelerations and decelerations in indoor sports (Manchado et al., 2013; Luteberget and Spencer, 2017; Puente et al., 2017). According information about required acceleration-based locomotion for each sport can easily be provided to sport scientists and coaches with the use of a complementary filtering technique, helping them to execute well-directed player replacements, adapt training programs, individualize recovery protocols and optimize athletic conditioning. In contrast to assessing the resulting vectors magnitude alone, this could lead to a deeper understanding of players' movements during training and competition indoors. A promising potential of IMU's is assumed in their ability to quantify locomotion but also to distinguish between distinct movement patterns. A number of studies in this relatively new field of interest has previously evaluated the validity of IMU-based variables, mainly PlayerLoad® and the resultant acceleration vector with respect to different movement patterns (Wundersitz et al., 2015a,b). Comparing peak acceleration values during walking, jogging and running a slight increase of the relative error was found for running (CV = 9.3%) compared to walking (CV = 6.5%) and jogging (CV = 7.5%) (Wundersitz et al., 2015b). In contrast no clear differences were observed when subjects performed 7 different team sport movements within a circuit, where CVs ranged from 3.7 to 6.9% only (Wundersitz et al., 2015b). When comparing validity of IMU-based accelerations during 3 different tackling tasks no differences in accuracy were apparent between two of the three movements (Wundersitz et al., 2015a). Overall, indications from literature do currently not suggest any obvious differences in validity of IMU's based on the performed movement itself. However, our results indicate that more detailed analysis in single movement planes seem to be possible and thereby might lead to according discrimination and quantification of movement patterns using IMU's in future by overcoming the restriction of the resulting acceleration vector only. Still, this study focused on the more general concurrent validity of sensor fusion algorithms under sport specific conditions and showed the potential of the applied complementary filtering technique to correctly estimate sports-related orientations in principle rather than for distinct movement patterns. Our findings therefore seem not sufficient enough to answer this question properly but should be taken into account for future research regarding the discrimination of movement patterns based on IMUs' output.

### Limitations

As a limitation of the study, the previously described misalignments between the tilt-corrected coordinate system and the reference axes of the MA system probably have an impact on accuracy in anterior-posterior and lateral direction, mainly affecting the relative error. Including only accelerometer and gyroscope data results in an orientation estimation relative to the direction of earth's gravity vector. Calculating absolute orientation with respect to the court's coordinates might be possible with the aid of magnetometer data and calibration trials, but further has to be validated. Ferromagnetic disturbances as they might occur during game days due to electronic sound systems around the court have to be considered for calculations. A certain amount of error has to be mentioned regarding the derivatives of MA system data, as the MA system directly measures displacement data not acceleration itself. Numerical differentiation of positional data can increase high-frequency noise of the MA data. Despite the attempt to partially dampen according inaccuracies, a potential influence on the criterion data has to be considered (Cole et al., 2014). However, MA system data are accepted as standard validation criterion for multiple player monitoring systems including acceleration estimates (Stevens et al., 2014; Vickery et al., 2014; Wundersitz et al., 2015a). Due to the short duration of recorded movement simulations we could not be aware of drifting phenomena as they might appear during longer trials. Drifting errors were not apparent during two recorded long-duration trials of 1.5 min, however this should be proved for a larger number of samples. When monitoring players during training or games another source of error might arise from enhanced vibrations of the device. For our experiments, the sensor has been fixed within a wooden frame to reduce unintentional whippings, which could occur when placing the sensor in a looser harness. This could presuppose different tuning parameters of the complementary filter as well as adaptions of the smoothing cut-off frequencies. Other microtechnological monitoring systems like GPS-devices showed a decrease in accuracy during short-distance or high-acceleration movements (Akenhead et al., 2014; Johnston et al., 2014). With a view to these findings it has to be mentioned that validity of IMUs in quantifying acceleration and deceleration efforts might also vary between specific movement patterns or intensities. This has not been part of this study, since we focused on the simulation of orientations as they might also occur during team sport activities rather than on actual movement performances. Therefore, our results are missing a conclusion about the advantages or disadvantages of IMU regarding the quantification of acceleration efforts during distinct movements.

## Conclusion

The findings of this study show that wearable tracking devices containing a MEMS-based sensor have a great potential to be applied also indoors as valid tool to determine accelerations and decelerations during of team sport specific movement including walking, running, jumping and change of direction simulations. The possibility to continuously analyze acceleration-values in horizontal and vertical planes broadens the field of player monitoring and comprehension of physical demands in indoor court-based sports. Coaches and sports scientists should be aware of the applied sensor fusion algorithm, its tuning parameters, correct smoothing technique and avoid analyzing raw accelerometer data to accurately determine the athlete's acceleration. Future research should aim to increase accuracy of accelerometer-derived data with the aid of magnetometers especially in x- and y-axes. Based on this, emphasis should be given to develop appropriate tools to detect an athlete's exact orientation on the court and the direction of performed movements in relation to the court's coordinate system. Discrimination between single movement patterns like backwards and forward movements, but also lateral motions and their proportion to each other should be investigated in future research and help to develop distinct activity profiles. Therefore, it would be critical to assess the validity and reliability of sensor fusion algorithms during actual performed different movement patterns and intensity zones. Further, numerical integration of acceleration values enables the calculation of according velocity which would lead to a deeper understanding of external loads in indoor team sports. For this purpose drift, which occurs due to the additive integration of noise within the IMU signal, has to be eliminated by appropriate algorithms. By providing comprehensive information about locomotion that exceed the restriction to resulting acceleration vectors, IMUs could become a meaningful tool for player monitoring in indoor team sports in future.

## Author contributions

MR, KR, and HM designed this study. Methodology was planned by MR, KR, and DG. MR and DG collected the data. MR analyzed and interpreted the data. AG provided funding acquisition and resources. MR drafted the manuscript. All authors revised the manuscript and approved the final version to be published.

### Conflict of interest statement

KR is scientific consultant for Adidas and is owner of a software company (ergonizer.com) for performance diagnostics. HM and MR receive research grants of Adidas. AG is scientific consultant for Adidas. This did not play any additional role in the study design, data collection and analysis, decision to publish, or preparation of the manuscript.
